# Supplementary material for: DENTIST—using long reads for closing assembly gaps at high accuracy
Source: Gigascience. 2022 Jan 25;11:giab100. doi: 10.1093/gigascience/giab100 (PMC8848313; doi:10.1093/gigascience/giab100)
Supplement: giab100_Response_to_Reviewer_Comments_Original_Submission [file giab100_response_to_reviewer_comments_original_submission.pdf]

Author's Response To Reviewer Comments

Close

We have uploaded a letter to the editor and a point by point response to all reviewer comments as two separate Word files.

Close
