# Supplementary material for: DENTIST—using long reads for closing assembly gaps at high accuracy
Source: Gigascience. 2022 Jan 25;11:giab100. doi: 10.1093/gigascience/giab100 (PMC8848313; doi:10.1093/gigascience/giab100)
Supplement: giab100_Reviewer_1_Report_Original_Submission — Edward Rice -- 10/14/2021 Reviewed [file giab100_reviewer_1_report_original_submission.pdf]

Reviewer Report

Title: DENTIST â€“ using long reads for closing assembly gaps at high accuracy

Version: Original Submission **Date:** 10/14/2021

Reviewer name: Edward Rice

Reviewer Comments to Author:

In this manuscript, the authors present a sophisticated method for closing gaps in assemblies, built around the knowledge that gaps usually occur in repetitive regions. They test their software against similar software with more realistic scenarios than previous studies, through the use of gaps from real assemblies of genomes that have other assemblies with fewer gaps, rather than randomly generated gaps. These tests convincingly demonstrate that this software is more sensitive and accurate than existing gap closers.

Given this increase in performance over existing software and the novelty of the methods, I recommend this manuscript for publication with some changes. I do have some concerns about the usability and maintainability of the software it describes, noted below, but most of the alternate options have similar issues, and the methodological advancements present in the manuscript merit publication.

1. The introduction seems to imply that the primary use of this software is for closing gaps in short-read assemblies where high-coverage long reads are not available due to cost. Although I do not have a statistic to back this up, it is my sense from recent genome assembly papers that long-read de novo assembly is much more the norm these days than short-read assembly. In my personal experience I have found that gap closing can sometimes greatly improve long-read assemblies as well, especially CLR assemblies of highly repetitive genomes. I recommend rewriting the introduction somewhat to make it clear that usage of this software is not limited to short-read assemblies, as these are becoming rarer and rarer.

2. I have some concerns about the maintainability of this code base, considering its size (>40k lines), language (D, which is not a common language in bioinformatics), and sparsity of comments in the code. Further, the use of non-standard dependencies and file formats may make it difficult to adapt the software to future advances in sequencing technology; for example, this package uses daligner to perform alignment, and so far as I can tell, daligner does not produce output in SAM format, so it may be difficult to switch to using another aligner in the future as the types of long reads available change. The fact that many of the dependencies are not maintained on bioconda is also concerning. The presence of integration tests is helpful. I apologize that this is probably not a particularly helpful comment as it's far too late to change any of these things, but still wanted to point them out.

3. I also have concerns about usability. The availability of a docker file and snakemake workflow for running this software and the thorough and mostly comprehensible documentation alleviate these concerns to some degree, but it still takes a significant amount of work to configure it for a specific cluster. The example run did not work out of the box without fixing some errors (see minor edits). To test on my own assembly, I had to edit one JSON file to choose the parameters for dentist itself, which required reading about the two ways to specify two required coverage parameters; one yaml file to

configure the workflow options; and one yaml file to make snakemake work with my cluster. In addition, not all clusters have singularity, so the lack of a conda package may be a problem for some potential users. The singularity image and snakemake workflow make its usability far better than PBJelly, which required actually editing the source code to make it work on my cluster with conda-installable versions of its dependencies, but it is still much worse than TGS-GapCloser, which only takes a single conda command to install with all dependencies and a single command to run, and no editing of configuration files.

Minor comments:

Abstract:

- "Here, we developed" -> "Here, we present"
- "Highly-accurate" â€" no hyphen
- "Short read assemblies" -> "short-read assemblies" (this occurs in several other places too throughout manuscript)
- Replace "right loci" with "correct loci"

Introduction:

- Page 3: "High contiguity, completeness, and accuracy… is fundamental" â€" change "is" to "are"
- Page 3: avoid parentheses inside other parentheses
- Page 3: I'm not sure I've ever heard of GenomicConsensus being used for gap closing, and cannot find any reference to it being used for this purpose with a quick scan of documentation. It must be capable of doing this, though, as you tested it alongside other gap closers. Could you explain this in the manuscript?

Results:

- Page 4: replace "right loci" with "correct loci"
- Page 4: say a little more about what makes DENTIST's "state-of-the-art" consensus module better than or different from existing consensus callers
- Page 5: "real life" to "real-life"
- Page 5: "high quality" to "high-quality"

Discussion:

- Page 9: "long read data" -> "long-read data"

Methods:

- Page 11: "genomic regions, where the number" â€" remove comma
- Page 12: "a common conflict are" to "a common conflict is"
- Page 12: "less than three reads" to "fewer than three reads"
- Page 14: "'copied' gaps from short read assembly" to "copied gaps from the short-read assembly"
- Page 14: remove quotation marks around "disassembled"

Software:

- The "small example" does not work out of the box as "dentist_v1.0.2.sif" is hard-coded into snakemake.yml but the image distributed with the example is v2.0.0.
- The "read-coverage" and "ploidy" options are listed as required (unless you're using "min-coverage-reads" and "max-coverage-reads", but they are not among the "important options" listed in the README under the "How to choose DENTIST parameters" subheading.
- In the more extensive list of command-line options, the description of the "read-coverage" option is

"this is used to provide good default values for -max-coverage-reads or -min-coverage-reads; both options are mutually exclusive." This tells the user how it is used by the program but gives the reader no explanation of how it should be chosen, which is important as it is one of the required options.
- The use of comments in dentist.json by putting double slashes in front of attribute strings is confusing and also not supported by the json specification. Dentist.json would be better in yaml format because:
a) YAML supports comments
b) YAML is easier to read by humans
c) YAML is used for the other two configuration files necessary to run the pipeline, so for consistency purposes it's best to have them all in the same format.

Level of Interest

Please indicate how interesting you found the manuscript: Choose an item.

Quality of Written English

Please indicate the quality of language in the manuscript: Choose an item.

Declaration of Competing Interests

Please complete a declaration of competing interests, considering the following questions:

- Have you in the past five years received reimbursements, fees, funding, or salary from an organisation that may in any way gain or lose financially from the publication of this manuscript, either now or in the future?
- Do you hold any stocks or shares in an organisation that may in any way gain or lose financially from the publication of this manuscript, either now or in the future?
- Do you hold or are you currently applying for any patents relating to the content of the manuscript?
- Have you received reimbursements, fees, funding, or salary from an organization that holds or has applied for patents relating to the content of the manuscript?
- Do you have any other financial competing interests?
- Do you have any non-financial competing interests in relation to this paper?

If you can answer no to all of the above, write 'I declare that I have no competing interests' below. If your reply is yes to any, please give details below.

I declare that I have no competing interests.

I agree to the open peer review policy of the journal. I understand that my name will be included on my report to the authors and, if the manuscript is accepted for publication, my named report including any attachments I upload will be posted on the website along with the authors' responses. I agree for my

report to be made available under an Open Access Creative Commons CC-BY license (http://creativecommons.org/licenses/by/4.0/). I understand that any comments which I do not wish to be included in my named report can be included as confidential comments to the editors, which will not be published.

Choose an item.

To further support our reviewers, we have joined with Publons, where you can gain additional credit to further highlight your hard work (see: https://publons.com/journal/530/gigascience). On publication of this paper, your review will be automatically added to Publons, you can then choose whether or not to claim your Publons credit. I understand this statement.

Yes Choose an item.
