# Supplementary material for: DENTIST—using long reads for closing assembly gaps at high accuracy
Source: Gigascience. 2022 Jan 25;11:giab100. doi: 10.1093/gigascience/giab100 (PMC8848313; doi:10.1093/gigascience/giab100)
Supplement: giab100_Reviewer_2_Report_Original_Submission — Leena Salmela -- 10/26/2021 Reviewed [file giab100_reviewer_2_report_original_submission.pdf]

Reviewer Report

Title: DENTIST â€“ using long reads for closing assembly gaps at high accuracy

Version: Original Submission **Date:** 10/26/2021

Reviewer name: Leena Salmela

Reviewer Comments to Author:

Overview:
The paper presents a new tool called DENTIST for closing gaps in short read assemblies using PacBio CLR data. Although new assemblies are nowadays most often done with PacBio HiFi data resulting in contiguous and accurate assemblies, closing the gaps of an existing short read assembly with long read data is a cost effecitve and therefore attractive alternative for species for which short read assemblies are already available. The new tool is shown to be more accurate than previous tools and of comparable sensitivity.
Suggestions for revision:
1) The authors should clearly indicate in the Introduction that their tool is tested on PacBio CLR reads. It would also be good to specify in the abstract that the reads were CLR reads and not HiFi reads.
2) In the Discussion, the authors recommend to "polish" the final gap closed assembly with Illumina reads. It would be interesting to see how much this improves the accuracy of gap closing. I would assume that the improvement on the gap sequences would be smaller than on other regions of the assembly because the gap sequences typically cover repetitive regions.
3) Last paragraph of section "Closing the gaps", page 14: DENTIST has three modes. Here it is indicated that the third mode (only use scaffolding information for conflict resolution and freely scaffold the contigs using long reads) would be the best mode for contig-only assemblies. It seems to me that also the second mode would be appropriate for this as it also closes gaps between scaffolds (or contigs in case of lack of scaffold information). Is this so?

Level of Interest

Please indicate how interesting you found the manuscript: Choose an item.

Quality of Written English

Please indicate the quality of language in the manuscript: Choose an item.

Declaration of Competing Interests

Please complete a declaration of competing interests, considering the following questions:

- Have you in the past five years received reimbursements, fees, funding, or salary from an organisation that may in any way gain or lose financially from the publication of this manuscript, either now or in the future?
- Do you hold any stocks or shares in an organisation that may in any way gain or lose financially from the publication of this manuscript, either now or in the future?
- Do you hold or are you currently applying for any patents relating to the content of the manuscript?
- Have you received reimbursements, fees, funding, or salary from an organization that holds or has applied for patents relating to the content of the manuscript?
- Do you have any other financial competing interests?
- Do you have any non-financial competing interests in relation to this paper?

If you can answer no to all of the above, write 'I declare that I have no competing interests' below. If your reply is yes to any, please give details below.

I declare that I have no competing interests.

I agree to the open peer review policy of the journal. I understand that my name will be included on my report to the authors and, if the manuscript is accepted for publication, my named report including any attachments I upload will be posted on the website along with the authors' responses. I agree for my report to be made available under an Open Access Creative Commons CC-BY license (http://creativecommons.org/licenses/by/4.0/). I understand that any comments which I do not wish to be included in my named report can be included as confidential comments to the editors, which will not be published.

Choose an item.

To further support our reviewers, we have joined with Publons, where you can gain additional credit to further highlight your hard work (see: https://publons.com/journal/530/gigascience). On publication of this paper, your review will be automatically added to Publons, you can then choose whether or not to claim your Publons credit. I understand this statement.

Yes Choose an item.
