# Supplementary material for: DENTIST—using long reads for closing assembly gaps at high accuracy
Source: Gigascience. 2022 Jan 25;11:giab100. doi: 10.1093/gigascience/giab100 (PMC8848313; doi:10.1093/gigascience/giab100)
Supplement: giab100_Reviewer_3_Report_Original_Submission — Ian Korf -- 10/31/2021 Reviewed [file giab100_reviewer_3_report_original_submission.pdf]

Reviewer Report

Title: DENTIST â€“ using long reads for closing assembly gaps at high accuracy

Version: Original Submission **Date:** 10/31/2021

Reviewer name: Ian Korf

Reviewer Comments to Author:

The paper by Ludwig et al demonstrates that DENTIST offers a substantial improvement in closing genomic assembly gaps. The paper is well written with a clear and concise style. I liked the way they approached the experiments with a combination of simulated and real data for both the assemblies and reads. Specifically, I applaud how they generated gaps where they actually happen. The figures are generally effective. The only exception to this is Figure 4 with the black background and inconsistent ordering of competing software. In addition to winning the bake-off against other software, they did a very useful analysis of read depth (figure 6) and resources used (table 2). These help future users plan their projects. From a code perspective, I like that they have put their code on github. I don't think they need to have the supplemental file of command line parameters, as anyone who wants to use the software is going to go to the github anyway, which has a much more comprehensive explanation of usage.

Level of Interest

Please indicate how interesting you found the manuscript: Choose an item.

Quality of Written English

Please indicate the quality of language in the manuscript: Choose an item.

Declaration of Competing Interests

Please complete a declaration of competing interests, considering the following questions:

- Have you in the past five years received reimbursements, fees, funding, or salary from an organisation that may in any way gain or lose financially from the publication of this manuscript, either now or in the future?
- Do you hold any stocks or shares in an organisation that may in any way gain or lose financially from the publication of this manuscript, either now or in the future?
- Do you hold or are you currently applying for any patents relating to the content of the manuscript?
- Have you received reimbursements, fees, funding, or salary from an organization that holds or has applied for patents relating to the content of the manuscript?
- Do you have any other financial competing interests?

- Do you have any non-financial competing interests in relation to this paper?

If you can answer no to all of the above, write 'I declare that I have no competing interests' below. If your reply is yes to any, please give details below.

I declare I have no competing interests

I agree to the open peer review policy of the journal. I understand that my name will be included on my report to the authors and, if the manuscript is accepted for publication, my named report including any attachments I upload will be posted on the website along with the authors' responses. I agree for my report to be made available under an Open Access Creative Commons CC-BY license (http://creativecommons.org/licenses/by/4.0/). I understand that any comments which I do not wish to be included in my named report can be included as confidential comments to the editors, which will not be published.

Choose an item.

To further support our reviewers, we have joined with Publons, where you can gain additional credit to further highlight your hard work (see: https://publons.com/journal/530/gigascience). On publication of this paper, your review will be automatically added to Publons, you can then choose whether or not to claim your Publons credit. I understand this statement.

Yes Choose an item.
