# Supplementary material for: DENTIST—using long reads for closing assembly gaps at high accuracy
Source: Gigascience. 2022 Jan 25;11:giab100. doi: 10.1093/gigascience/giab100 (PMC8848313; doi:10.1093/gigascience/giab100)
Supplement: giab100_Supplemental_Files [file giab100_supplemental_files.zip › PointByPointResponse.docx]

**Point by point response**

**Reviewer reports:**

**Reviewer #1:**

In this manuscript, the authors present a sophisticated method for closing gaps in assemblies, built around the knowledge that gaps usually occur in repetitive regions. They test their software against similar software with more realistic scenarios than previous studies, through the use of gaps from real assemblies of genomes that have other assemblies with fewer gaps, rather than randomly generated gaps. These tests convincingly demonstrate that this software is more sensitive and accurate than existing gap closers.

Given this increase in performance over existing software and the novelty of the methods, I recommend this manuscript for publication with some changes. I do have some concerns about the usability and maintainability of the software it describes, noted below, but most of the alternate options have similar issues, and the methodological advancements present in the manuscript merit publication.

We thank the reviewer for carefully testing DENTIST and the suggestions to increase its usability and user-friendliness.

1. The introduction seems to imply that the primary use of this software is for closing gaps in short-read assemblies where high-coverage long reads are not available due to cost. Although I do not have a statistic to back this up, it is my sense from recent genome assembly papers that long-read de novo assembly is much more the norm these days than short-read assembly. In my personal experience I have found that gap closing can sometimes greatly improve long-read assemblies as well, especially CLR assemblies of highly repetitive genomes. I recommend rewriting the introduction somewhat to make it clear that usage of this software is not limited to short-read assemblies, as these are becoming rarer and rarer.

We thank the reviewer for raising this point. Indeed, the assembly pipelines used by the VGP or Bat1K include gap filling as a final step for long read based assemblies. We now included that in the introduction:

“Furthermore, gap filling is also one of the final steps in state-of-the-art pipelines to generate high-quality long read-based assemblies [1].”

We also made clearer that long read sequencing is the norm these days:

“These reads are often longer than genomic repeats, which enables the assembly of highly-complete and highly-contiguous genomes, and are therefore increasingly used for *de novo* assembly [1-3,7].“

However, we would like to mention that hundreds of short read assemblies exist, exemplified by the publications from the Zoonomia and B10K project, and therefore now added in the introduction:

“... genomes of numerous species have been sequenced with short read technologies such as Illumina instruments, illustrated by the Zoonomia and B10K project that generated new assemblies for 131 mammals and 267 birds, respectively [5,6]”

2. I have some concerns about the maintainability of this code base, considering its size (>40k lines), language (D, which is not a common language in bioinformatics), and sparsity of comments in the code. Further, the use of non-standard dependencies and file formats may make it difficult to adapt the software to future advances in sequencing technology; for example, this package uses daligner to perform alignment, and so far as I can tell, daligner does not produce output in SAM format, so it may be difficult to switch to using another aligner in the future as the types of long reads available change. The fact that many of the dependencies are not maintained on bioconda is also concerning. The presence of integration tests is helpful. I apologize that this is probably not a particularly helpful comment as it's far too late to change any of these things, but still wanted to point them out.

We thank the reviewer for the careful consideration of usability. As suggested by the reviewer, we have created a Conda package for DENTIST. The tool can now be run by three different ways (compiled binaries, Conda package and Singularity container), which we hope will work in most setups.

Using the systems we have available at two institutes, installation of DENTIST and running the test examples worked without problems. We have also improved the readability and understandability of the source code by adding more comments, as suggested.

We agree with the reviewer that D is not common in bioinformatics. Still, in our humble opinion, it is a remarkable programming language that we would like to promote.

Furthermore, we agree that the intermediate formats lack interoperability with other alignment software and will address this issue in a future release of DENTIST. In the current implementation, the tools were chosen for their good performance and a low disk space requirements of the file formats.

3. I also have concerns about usability. The availability of a docker file and snakemake workflow for running this software and the thorough and mostly comprehensible documentation alleviate these concerns to some degree, but it still takes a significant amount of work to configure it for a specific cluster. The example run did not work out of the box without fixing some errors (see minor edits). To test on my own assembly, I had to edit one JSON file to choose the parameters for dentist itself, which required reading about the two ways to specify two required coverage parameters; one yaml file to configure the workflow options; and one yaml file to make snakemake work with my cluster. In addition, not all clusters have singularity, so the lack of a conda package may be a problem for some potential users. The singularity image and snakemake workflow make its usability far better than PBJelly, which required actually editing the source code to make it work on my cluster with conda-installable versions of its dependencies, but it is still much worse than TGS-GapCloser, which only takes a single conda command to install with all dependencies and a single command to run, and no editing of configuration files.

We are grateful for the thorough tests that the reviewer performed, which revealed new problems that we were not aware of. As mentioned above, we now provide a Conda package, which enables those users that lack Singularity on their HPC system to run DENTIST. We also corrected the version specifier in the example.

Minor comments:

Abstract:

- "Here, we developed" -> "Here, we present"

- "Highly-accurate" — no hyphen

- "Short read assemblies" -> "short-read assemblies" (this occurs in several other places too throughout manuscript)

- Replace "right loci" with "correct loci"

All these issues are fixed in the revised manuscript.

Introduction:

- Page 3: "High contiguity, completeness, and accuracy… is fundamental" — change "is" to "are"

- Page 3: avoid parentheses inside other parentheses

- Page 3: I'm not sure I've ever heard of GenomicConsensus being used for gap closing, and cannot find any reference to it being used for this purpose with a quick scan of documentation. It must be capable of doing this, though, as you tested it alongside other gap closers. Could you explain this in the manuscript?

“GenomicConsensus” refers to the PacBio package that provides the variantCaller tool that applies either the Quiver or Arrow algorithm. We now clarified this by writing:

“PacBio GenomicConsensus (PacBio GC,<https://github.com/PacificBiosciences/GenomicConsensus/>) comprising the Arrow and Quiver algorithm implemented in the variantCaller tool”

We ran GenomicConsensus by calling the executable ‘variantCaller --algorithm=best’, which automatically selects the most appropriate method depending on the PacBio chemistry used to generate the long reads. As this information was previously only contained in the Supplementary Table 3, we now clarified this in the methods as well.

Results:

- Page 4: replace "right loci" with "correct loci"

- Page 4: say a little more about what makes DENTIST's "state-of-the-art" consensus module better than or different from existing consensus callers

Daccord uses a local De Bruijn graph to construct a consensus sequence, which was shown to have increased accuracy over other methods (Tischler & Myers, 2017). We revised the respective sentence to

“The cleaned up alignment is used as input for *daccord* [16], which uses a local de Bruijn graph-based approach to compute an accurate consensus based on the selected reference read.”

- Page 5: "real life" to "real-life"

- Page 5: "high quality" to "high-quality"

Discussion:

- Page 9: "long read data" -> "long-read data"

Methods:

- Page 11: "genomic regions, where the number" — remove comma

- Page 12: "a common conflict are" to "a common conflict is"

- Page 12: "less than three reads" to "fewer than three reads"

- Page 14: "'copied' gaps from short read assembly" to "copied gaps from the short-read assembly"

- Page 14: remove quotation marks around "disassembled"

All other smaller points have also been corrected in the revised manuscript. We thank the reviewer for pointing these out.

Software:

- The "small example" does not work out of the box as "dentist_v1.0.2.sif" is hard-coded into snakemake.yml but the image distributed with the example is v2.0.0.

We are thankful for the careful trial of our software. We fixed the mentioned version number.

- The "read-coverage" and "ploidy" options are listed as required (unless you're using "min-coverage-reads" and "max-coverage-reads", but they are not among the "important options" listed in the README under the "How to choose DENTIST parameters" subheading.

The reviewer is correct that these are important options. Therefore, we added detailed descriptions of these parameters to the configuration guide at GitHub (https://github.com/a-ludi/dentist#configuration):

- --read-coverage: This is the preferred way of providing values to --max-coverage-reads, --max-improper-coverage-reads and --min-coverage-reads. See below how their values are derived from --read-coverage.

Ideally, the user provides the haploid read coverage, which can be inferred using a histogram of the alignment coverage across the assembly. Alternatively, the average raw read coverage can be used, which is the number of base pairs in the reads divided by the number of base pairs in the assembly.

- --ploidy: Combined with --read-coverage, this parameter is the preferred way of providing --min-coverage-reads.

We use the Wikipedia definition of ploidy, as “the number of complete sets of chromosomes in a cell” (<https://en.wikipedia.org/wiki/Ploidy>)

Furthermore, we included details on how the derived parameters are computed (see configuration guide at GitHub, parameters –max-coverage-reads and –min-coverage-reads).

- In the more extensive list of command-line options, the description of the "read-coverage" option is "this is used to provide good default values for -max-coverage-reads or -min-coverage-reads; both options are mutually exclusive." This tells the user how it is used by the program but gives the reader no explanation of how it should be chosen, which is important as it is one of the required options.

We adjusted the help text of DENTIST’s command-line options to:

“This is used to provide good default values for --max-coverage-reads (mask-repetitive-regions) or --min-coverage-reads (validate-regions); --read-coverage and --*-coverage-reads are mutually exclusive. Ideally, the user provides the haploid read coverage which, for example, may be inferred using a histogram of the alignment coverage across the genome. Alternatively, the average raw read coverage can be used which is the number of base pairs in the reads divided by the number of base pairs in the assembly.”

- The use of comments in dentist.json by putting double slashes in front of attribute strings is confusing and also not supported by the json specification. Dentist.json would be better in yaml format because:

a) YAML supports comments

b) YAML is easier to read by humans

c) YAML is used for the other two configuration files necessary to run the pipeline, so for consistency purposes it's best to have them all in the same format.

Following the reviewer’s suggestion, we now allow the configuration in YAML format. We also converted the example configuration files to YAML.

**Reviewer #2:**

Overview:

The paper presents a new tool called DENTIST for closing gaps in short read assemblies using PacBio CLR data. Although new assemblies are nowadays most often done with PacBio HiFi data resulting in contiguous and accurate assemblies, closing the gaps of an existing short read assembly with long read data is a cost effecitve and therefore attractive alternative for species for which short read assemblies are already available. The new tool is shown to be more accurate than previous tools and of comparable sensitivity.

We thank the reviewer for these kind words and the comments.

Suggestions for revision:

1) The authors should clearly indicate in the Introduction that their tool is tested on PacBio CLR reads. It would also be good to specify in the abstract that the reads were CLR reads and not HiFi reads.

As suggested, we changed the respective abstract sentence to

“... using simulated or real PacBio continuous long reads, we show that DENTIST ... ”

2) In the Discussion, the authors recommend to "polish" the final gap closed assembly with Illumina reads. It would be interesting to see how much this improves the accuracy of gap closing. I would assume that the improvement on the gap sequences would be smaller than on other regions of the assembly because the gap sequences typically cover repetitive regions.

The Vertebrate Genome Project assembly pipeline (Rhie *et al.* 2021) recommends polishing with 10X linked read data, which can also be mapped many repetitive regions. Rhie *et al.* show that the best consensus quality achievable with PacBio CLR reads is Q30–35. This will also hold for DENTIST, and in regions of lower read coverage, the consensus may be less accurate. With polishing Rhie *et al.* demonstrate that the quality consistently increases to Q40 (99.99% accuracy). Therefore, to avoid users skipping the polishing step because DENTIST’s base accuracy is higher than other methods, we explicitly discuss the importance of polishing in the Discussion and we mention that DENTIST’s more accurate consensus will facilitate the mapping of short read data.

3) Last paragraph of section "Closing the gaps", page 14: DENTIST has three modes. Here it is indicated that the third mode (only use scaffolding information for conflict resolution and freely scaffold the contigs using long reads) would be the best mode for contig-only assemblies. It seems to me that also the second mode would be appropriate for this as it also closes gaps between scaffolds (or contigs in case of lack of scaffold information). Is this so?

The reviewer is absolutely correct. We now clarified this by writing:

“The second and third mode enables DENTIST to also improve contig-only assemblies.“

**Reviewer #3:**

The paper by Ludwig et al demonstrates that DENTIST offers a substantial improvement in closing genomic assembly gaps. The paper is well written with a clear and concise style. I liked the way they approached the experiments with a combination of simulated and real data for both the assemblies and reads. Specifically, I applaud how they generated gaps where they actually happen. The figures are generally effective.

We thank the reviewer for these kind words and the comments.

The only exception to this is Figure 4 with the black background and inconsistent ordering of competing software.

We have changed Figure 4 to provide a larger spacing between the black blocks. The assembly gap position is now shown in blue and the ordering of the methods is now consistently alphabetically. We thank the reviewer for noticing that.

In addition to winning the bake-off against other software, they did a very useful analysis of read depth (figure 6) and resources used (table 2). These help future users plan their projects. From a code perspective, I like that they have put their code on github. I don't think they need to have the supplemental file of command line parameters, as anyone who wants to use the software is going to go to the github anyway, which has a much more comprehensive explanation of usage.

We agree with the reviewer, but we thought that having an up-to-date description of the parameters as a Supplementary Table would be helpful for readers that do not want to search for this on the GitHub page. If the reviewer agrees, we would prefer to keep the table in the Supplement.
